# Electroconvulsive therapy modulates Fronto-temporal functional connectivity in adolescents with depression and suicidal ideation

**DOI:** 10.3389/fnimg.2026.1811399

**Published:** 2026-04-10

**Authors:** Xiaolu Chen, Jianmei Chen, Ming Ai, Su Hong, Linxi Dai, Xiaoshan Shen, Li Kuang

**Affiliations:** 1 Department of Psychiatry, Key Laboratory of Major Brain Disease and Aging Research (Ministry of Education), The First Affiliated Hospital of Chongqing Medical University, Chongqing, China; 2 Psychiatric Center, The First Affiliated Hospital of ChongQing Medical University, Chongqing, China; 3 Department of the First Clinical Medicine, Chongqing Medical University, Chongqing, China

**Keywords:** electroconvulsive therapy, functional magnetic resonance imaging, major depressive disorder, region of interest, resting-state functional connectivity, suicidal ideation

## Abstract

**Objective:**

We aimed to investigate changes in whole-brain functional connectivity (FC) before and after electroconvulsive therapy (ECT) in adolescents with major depressive disorder (MDD) and suicidal ideation (SI).

**Methods:**

Forty-nine adolescents with MDD and SI were enrolled, and resting-state functional magnetic resonance imaging (rs-fMRI) was performed at baseline and after ECT for each patient. Forty healthy controls (HCs) were scanned only at baseline. Region-of-interest (ROI)-based whole-brain FC analyses were used, with the left superior frontal gyrus (L-SFG) and right superior temporal gyrus (R-STG) as seed regions.

**Results:**

Compared with HCs, MDD patients at baseline showed decreased FC between R-STG and left inferior occipital gyrus (L-IOG), and between L-SFG and right anterior cingulate gyrus (R-ACG). After ECT, MDD patients showed reduced FC between R-STG and right middle temporal gyrus (R-MTG), increased FC between L-SFG and right middle frontal gyrus (R-MFG), and decreased FC between L-SFG and right superior occipital gyrus (R-SOG)/right superior frontal gyrus (R-SFG). Pearson’s correlation found that post-ECT Hamilton Depression Rating Scale-17 (HAMD-17) scores were negatively correlated with FC between R-STG and L-IOG.

**Conclusion:**

Abnormal FC in the frontal-cingulate and frontal-temporal circuits may be a potential neurobiological basis of depressive and suicidal symptoms in adolescents. ECT may improve these symptoms by modulating FC in these key brain regions.

## 1 Introduction

Major Depressive Disorder (MDD) is widely recognised as one of the most critical global mental health issues, as it significantly increases the risk of suicide (American Psychiatric Association, 2011). As a key precursor to suicide, MDD is strongly associated with the development of suicidal ideation (SI) and suicidal behaviors. Specifically, SI is defined as “thoughts regarding death, dying, suicide plans, or a desire for death (Miller et al., 2018; Levi-Belz et al., 2019),” and it serves as a robust predictor of death by suicide.

Electroconvulsive therapy (ECT) is considered to be effective for MDD (UK ECT Review Group, 2003), especially for MDD with SI or suicidal attempt (SA) (McCall, 2016). For its use on adolescents, multiple studies have demonstrated that ECT is effective for adolescents with depression who exhibit SI or suicidal behavior (Puffer et al., 2016; Mitchell et al., 2018). One possible reason ECT is effective in reducing SI and suicidal behaviors lies in its impact on the functioning of brain regions associated with emotional regulation and behavioural control.

Resting state functional connectivity (RSFC) operates by detecting temporal correlations in neuronal activity across anatomically distinct brain regions during the resting state (Friston, 1994). It is now widely applied in psychiatric research, with common analytical approaches including seed-based functional connectivity(FC). Seed-based FC is a prevalent approach within RSFC studies. This method involves designating a region of interest (ROI), followed by calculating the temporal correlations between this ROI and voxels across the entire brain. This process determines whether the seed exhibits specific connections with the whole brain, through this method, we understand the relationship between psychiatric symptoms and FC in mental disorders. Therefore, we consider that ROI-based FC analysis could also serve as a research tool for studying the condition in adolescents with depression.

The prefrontal cortex is considered to be significantly involved in the neurobiological mechanisms underlying depressive and suicidal symptoms. Studies have shown significant reductions in prefrontal volume among depressed individuals at high suicide risk (Greicius et al., 2007; Wagner et al., 2011; Zhang et al., 2020). Beyond the prefrontal regions, the temporal lobes are also implicated in the neurobiological mechanisms of depression with suicidal features. Studies (Gosnell et al., 2016; McLellan et al., 2018) have demonstrated pronounced temporal lobe volume reduction in MDD patients with suicide attempts than in those without. Both structural and local brain functional studies have revealed that the frontal and temporal lobes exhibit increased involvement in adolescents with depression who have SI. Specifically, structural and functional abnormalities in the left superior frontal gyrus (L-SFG) and right superior temporal gyrus (R-STG) correlate closely with depressive symptoms and suicidal ideation. Therefore, in the present study, we designated the L-SFG and R-STG as ROIs to explore abnormal FC in adolescent MDD patients with SI and investigate the neurobiological mechanisms underlying ECT efficacy through a whole-brain FC analysis.

In the present study, we examined FC among adolescents with MDD and SI using L-SFG and R-STG as ROI. Our study aims to explore the abnormal FC in adolescent MDD patients with SI and the neurobiological mechanisms underlying ECT efficacy through a whole-brain FC analysis model based on ROIs.

## 2 Materials and methods

### 2.1 Subjects

The study included 40 healthy controls (HCs) and 49 adolescents (12–18 years) with major depressive disorder (MDD) and clinically significant suicidality. Diagnosis was confirmed by two psychiatrists using the Mini International Neuropsychiatric Interview for Children and Adolescents (MINI-KID) (Sheehan et al., 2010). Inclusion criteria were: (1) a Hamilton Depression Rating Scale-17 (HAMD-17) score ≥ 17; (2) no prior exposure to ECT; and (3) severe suicidality indicated by a Beck Scale for Suicide Ideation (BSSI) score ≥ 11 within the past 7 days. Exclusion criteria were: (1) major neurological or severe medical illness, substance/alcohol abuse, or structural brain abnormalities; (2) implanted electronic/metal devices incompatible with MRI; and (3) excessive head motion (translation > 2.5 mm or rotation > 2.5°). Healthy controls were matched to patients by age, sex, and education, and had no current/past psychiatric disorder, no family history of major psychiatric illness, no neurological/severe medical disease, no psychotropic medication or psychotherapy within the preceding 6 months, and no MRI contraindications or excessive head motion.

The investigation process was authorized by the Human Research and Ethics Committee of Chongqing Medical University’s First Affiliated Hospital (no. 2017-157). Teenagers, together with their caretakers, granted signed informed consent. The study was registered with the Chinese Clinical Trial Registry, the ID of which is ChiCTR2200064527.

The study showed that 100% of patients with MDD were treated with sertraline, an antidepressant, during the trial, and 10.2% of them were concomitantly administered aripiprazole, an antipsychotic. In contrast, no psychotropic medications were used in the HC group in the 6 months prior to the study. It is critical to distinguish the specific effects of the intervention from concomitant medical treatments when evaluating clinical trial data. The baseline FC differences observed between the MDD and HC groups may be potentially influenced by pharmacological effects. Although the duration of medication exposure in the study was relatively short and antidepressants are generally considered to have no immediate therapeutic effects within such a short period, the potential impact of these medications on brain functional connectivity cannot be excluded, which is a notable limitation of the present study.

### 2.2 Clinical assessment

Participants underwent assessments of both the HAMD-17 score and the BSSI score (Hamilton, 1960; Beck et al., 1979) at both baseline and after ECT. The Chinese versions of these scales have strong validity and reliability (Zhao and Zheng, 1992; Li et al., 2010).

### 2.3 Electroconvulsive therapy

Modified bilateral ECT was administered via the Thymatron DGx device (Somatics, LLC, Lake Bluff, Illinois, USA) at Chongqing Medical University’s First Affiliated Hospital. The treatment protocol was as follows: the first three sessions were conducted consecutively on a daily basis, followed by alternate-day treatments (with weekends excluded), with every participant completing 8 sessions during the study period. The initial stimulus dose was determined by the age × 0.5% formula, with subsequent dose adjustments based on seizure duration: the epileptiform seizure lasted <25 s, and then the energy was increased by 5% for the next session. The primary induction agents used were propofol and succinylcholine, which were dosed at 1.5–2 mg/kg and 0.5–1 mg/kg, respectively. Every patient maintained their antidepressant medications during treatment: sertraline in all patients (100%). Antipsychotics (aripiprazole) were used in 5 patients (10.2%).

For ethical reasons, adolescents with severe suicidal ideation required timely and effective intervention; therefore, a separate non-ECT control group (e.g., a medication-only group) was not established. Accordingly, the potential confounding effects of concomitant medication use, the passage of time, and repeated rs-fMRI exposure on FC changes cannot be fully excluded. This issue is further discussed in the Limitations section.

### 2.4 Acquisition of rs-fMRI data

A 3.0 T GE Signa HDxt scanner (GE Healthcare, USA) was used to conduct magnetic resonance imaging, featuring a head coil that has eight channels. Throughout the scan, all the participants were instructed to remain awake while keeping their eyelids closed and to minimize intentional mental activity. Participants were monitored by the operator, and none reported falling asleep during the imaging session. To reduce scanner noise, protective earplugs were provided, and foam padding was used to minimize head motion. Resting-state functional images were acquired using an echo-planar imaging (EPI) sequence with the following parameters: repetition time 2,000 ms; field of view 240 mm × 240 mm; echo time 40 ms; matrix size 64 × 64; flip angle 90°; 33 axial slices without gaps (slice thickness 4.0 mm); total acquisition time 8 min; and 240 volumetric datasets obtained. For the anatomical coregistration of the functional data, high-resolution T1-weighted structural images were obtained (repetition time, 24 ms; echo time, 9 ms; field of view, 240 mm × 240 mm; matrix, 256 × 256; flip angle, 90°; and slice thickness, 1.0 mm without gaps).

### 2.5 Image preprocessing

DPARSF (Data Processing Assistant for Resting-State fMRI, Version 4.3) (Yan and Zang, 2010), based on SPM12, was used for preprocessing in MATLAB (MathWorks Inc., Natick, MA, USA). The first 10 volumes were discarded. Subsequent steps included slice-timing correction, head-motion realignment, spatial normalization to MNI space (resampled to 3 × 3 × 3 mm^3^), and nuisance regression using 24 motion parameters (Power et al., 2012) and signals from white matter and cerebrospinal fluid. Linear trends were removed, and the data were temporally filtered using a 0.01–0.08 Hz band-pass filter to reduce physiological noise and low-frequency drift.

### 2.6 Functional connectivity analysis

Seed region selection. In the present study, a hypothesis-driven seed selection strategy was adopted. Based on previous findings implicating the L-SFG and R-STG in depressive symptoms and suicidal ideation in adolescents with MDD, this approach allowed us to test our *a priori* hypothesis in a targeted manner. Data-driven approaches, such as independent component analysis or whole-brain voxel-wise analyses, were not included in the current study, partly because the modest sample size might have limited the robustness of such exploratory analyses and increased the complexity of multiple-comparison control. We acknowledge that hypothesis-driven seed selection may introduce inherent selection bias. Future studies with larger samples may combine hypothesis-driven and data-driven approaches to provide a more comprehensive characterization of whole-brain network alterations. Seed ROIs were defined anatomically using the Automated Anatomical Labeling (AAL) atlas in MNI space. The AAL parcels corresponding to the L-SFG and R-STG were extracted, and the mean BOLD time series was averaged across all voxels within each parcel. Calculation of functional connectivity. The mean time series of the L-SFG and R-STG seed parcels were extracted from the preprocessed data. Pearson correlation analysis was performed between the mean time series of each seed region and the time series of every other voxel in the whole brain to generate seed-based FC maps for each participant. Finally, a Fisher’s r-to-z transformation was applied to convert the correlation coefficients into *z*-values to improve normality for subsequent statistical analyses.

### 2.7 Statistical analysis

Statistical analyses were performed using SPSS version 26.0 (IBM Corp., Armonk, NY, USA). Continuous variables were expressed as mean ± standard deviation (SD). The normality of data distribution was assessed using the Shapiro–Wilk test. For demographic and clinical data, group differences were examined using two-sample *t*-tests (for normally distributed data) or Mann–Whitney *U* tests (for non-normally distributed data). Categorical variables were compared using the chi-square test. For fMRI data, statistical analyses were performed using DPABI software. Baseline comparison: A two-sample *t*-test was employed to identify voxel-wise FC differences between MDD patients and HCs, with sex, age, education level, and mean FD included as covariates. Longitudinal comparison: A paired *t*-test was used to examine FC alterations within the MDD group before and after ECT. All statistical maps were corrected for multiple comparisons using Gaussian Random Field (GRF) theory (voxel-level *p* < 0.001, cluster-level *p* < 0.05, two-tailed). Finally, mean FC values were extracted from the brain regions showing significant differences. Pearson correlation analysis (or Spearman correlation analysis for non-normal data) was conducted to evaluate the associations between these FC values and clinical symptom scores (HAMD-17/BSSI). A *p*-value < 0.05 was considered statistically significant.

## 3 Results

### 3.1 Statistical results for general data

See Tables 1 and 2.

**Table 1:** Demographics and baseline clinical features of the participants.

Characteristic	HC (*n* = 40)	MDD (*n* = 49)	*t*/*χ*^2^	*p*
Age, mean (SD), y	14.73 (1.65)	15.17 (1.58)	−1.292^a^	0.200
Sex (male/female)	10/30	15/34	0.122^b^	0.727
Education years, mean (SD), y	8.93 (1.58)	8.78 (1.45)	0.469^a^	0.640
First onset age, mean (SD), y	–	14.46 (1.31)	–	–
Duration of illness, mean (SD), months	–	5.37 (4.45)	–	–
HAMD-17, mean (SD) [Range]	2.00 (1.20) [0–5]	23.97 (2.75) [19–32]	−50.402^a^	<0.001
BSSI, mean (SD) [Range]	1.58 (1.43) [0–6]	21.65 (3.90) [14–28]	−33.367^a^	<0.001
Medication status				
Antidepressants, *n* (%)	–	49 (100%)^c^	–	–
Antipsychotics, *n* (%)	–	5 (10.2%)^c^	–	–

SD, Standard Deviation; y, years.

a Two-sample *t*-test.

b Chi-square test.

c Proportion of patients taking medications within the MDD group.

**Table 2:** Clinical symptom severity before and after ECT.

Characteristic	pre-ECT	post-ECT	*t*	*p*
HAMD-17, mean (SD) [Range]	23.97 (2.75) [19–32]	10.51 (2.29) [6–16]	33.119^a^	<0.001
BSSI, mean (SD) [Range]	21.65 (3.90) [14–28]	5.17 (1.93) [2–9]	34.790^a^	<0.001

a Paired *t*-test. Values in the “pre-ECT” column correspond to the baseline values of the MDD group in Table 1.

### 3.2 FC differences between HC and MDD groups at baseline

**Figure 1:**
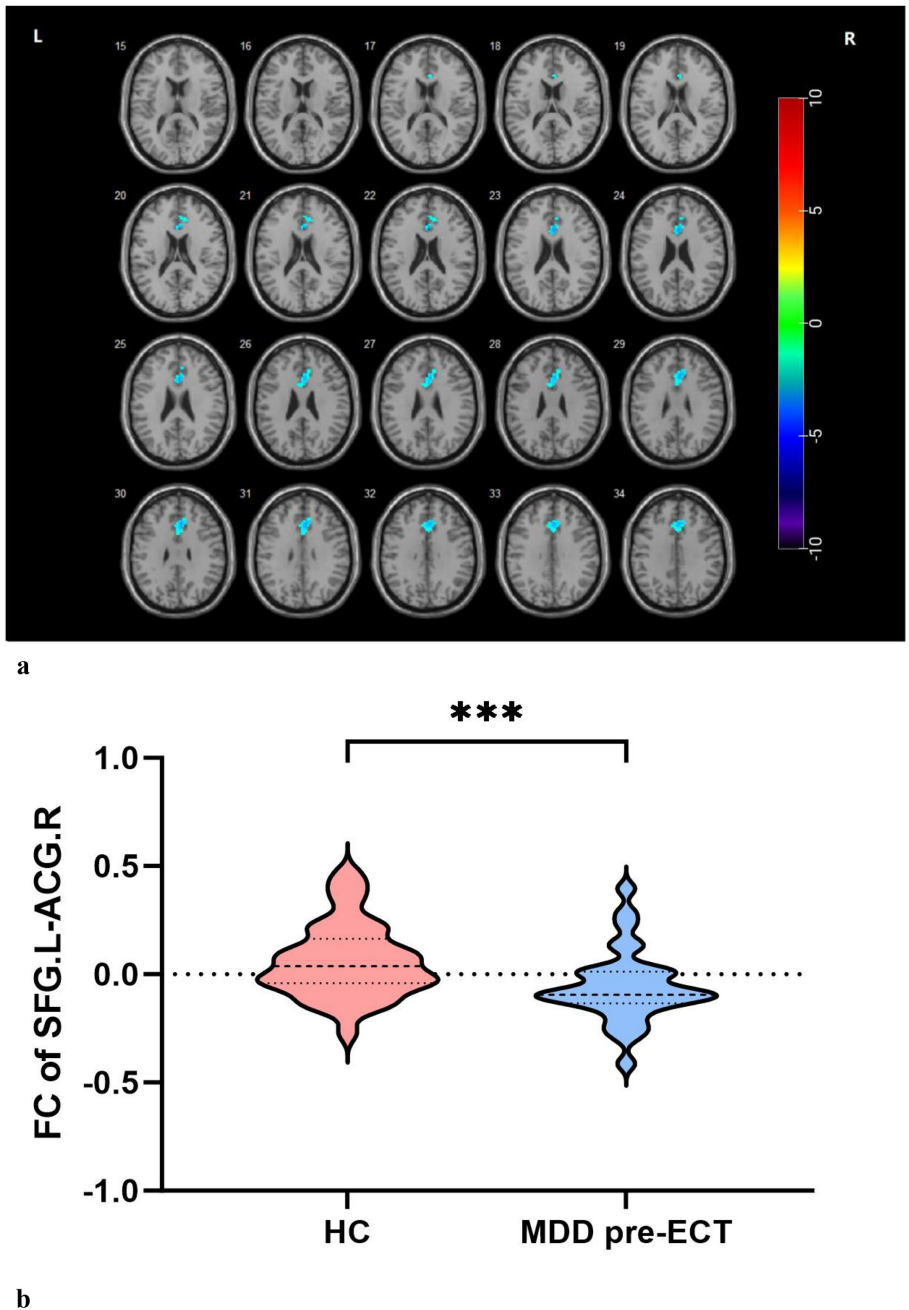
**(a, b)** Decreased FC between L-SFG and R-ACG in MDDs at baseline compared with HCs.

Seed-based FC revealed that when using the right superior temporal gyrus (R-STG) as the ROI, the MDD group exhibited decreased FC in the R-STG and left inferior occipital gyrus (L-IOG) compared to the HC group at baseline (Table 3, Figure 1).

**Table 3:** Changed FC in brain regions between MDDs and HCs (seed 1: R-STG).

Brain region	MNI peak coordinates			*T* value	Cluster size
	*X*	*Y*	*Z*		
L-IOG	−24	−96	−9	−4.2832	55

### 3.3 FC differences in the MDD group following ECT

**Figure 2:**
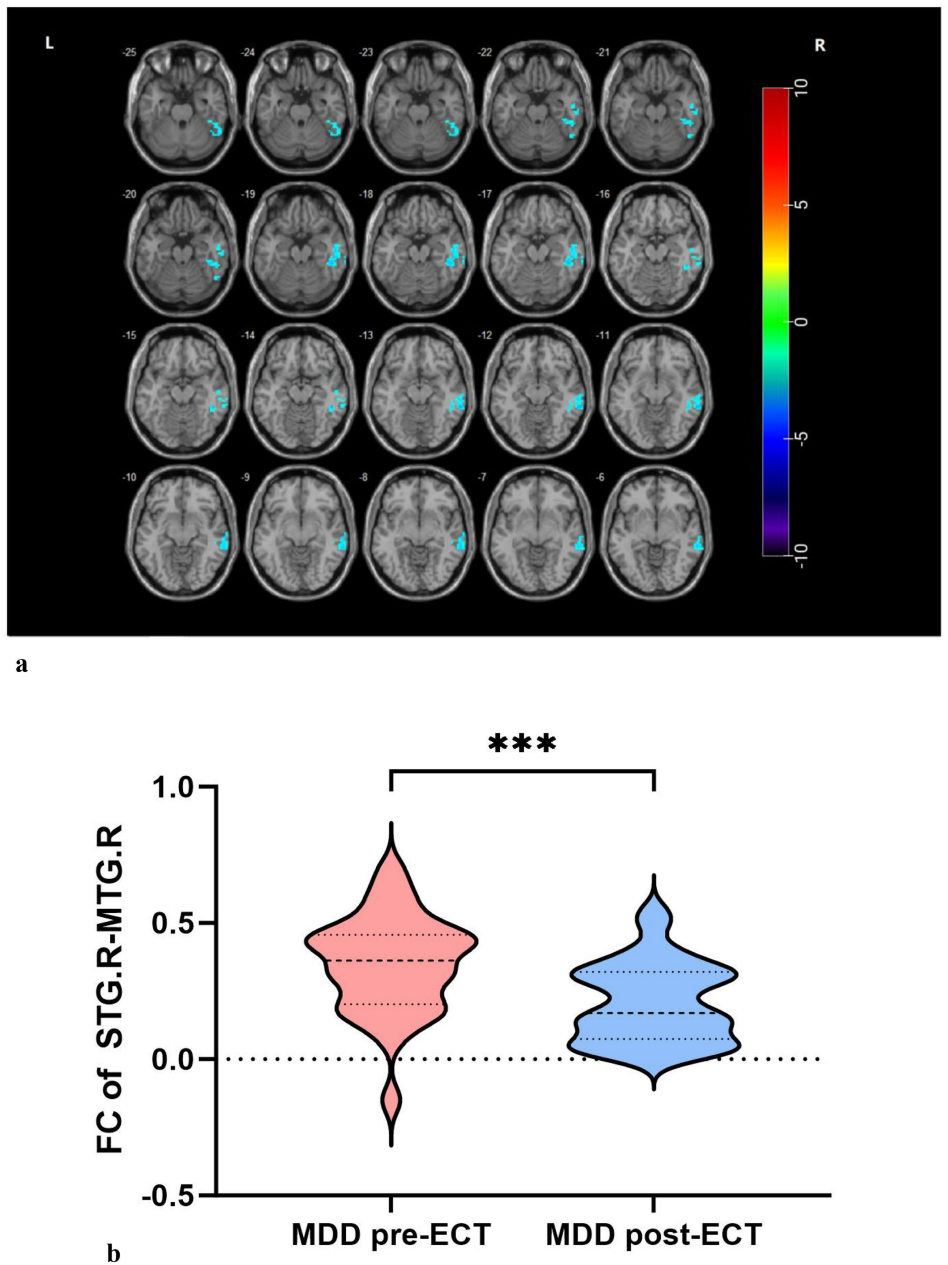
**(a, b)** Decreased FC between R-STG and R-MTG in MDDs after ECT.

Compared with pre-ECT, FC between the ROI and whole-brain voxels showed changes in the MDD group post-ECT. Analysis using the R-STG as the ROI revealed decreased FC between R-STG and the right middle temporal gyrus (R-MTG) following ECT (Table 4, Figure 2).

**Table 4:** Changed FC in MDDs after ECT (seed 1: R-STG).

Brain region	MNI peak coordinates			*T* value	Cluster size
	*X*	*Y*	*Z*		
R-MTG	63	−39	−12	−4.0633	202

**Figure 3:**
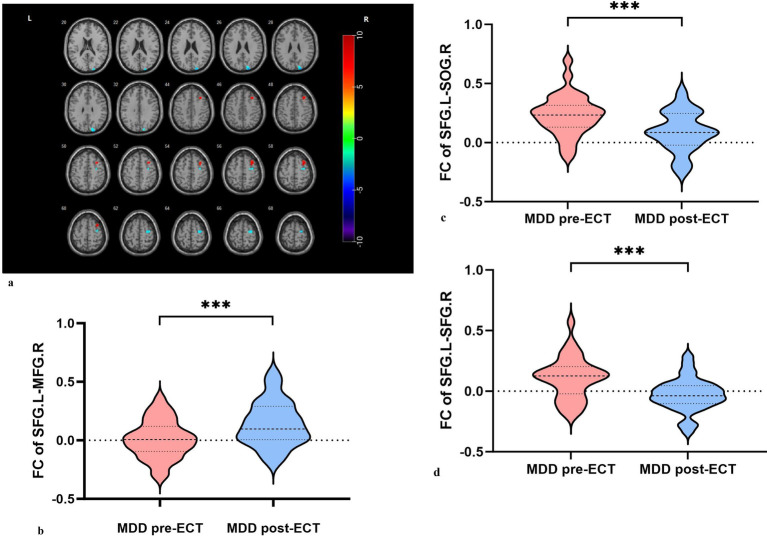
**(a)** Decreased FC between L-SFG and R-SOG, between L-SFG and R-SFG, increased FC between L-SFG and R-MFG in MDDs after ECT. **(b)** Increased FC was found between L-SFG and R-MFG in MDDs after ECT. **(c)** Decreased FC was found between L-SFG and R-SOG in MDDs after ECT. **(d)** Decreased FC was found between L-SFG and R-SFG in MDDs after ECT.

Analysis using the L-SFG as ROI revealed increased FC between L-SFG and right middle frontal gyrus (R-MFG), while FC between L-SFG and right superior occipital gyrus (R-SOG) and R-SFG was decreased (Table 5, Figure 3).

**Table 5:** Changed FC in MDDs after ECT (seed 2: L-SFG).

Brain region	MNI peak coordinates			*T* value	Cluster size
	*X*	*Y*	*Z*		
R-MFG	36	12	60	4.0115	57
R-SOG	21	−93	27	−4.7353	46
R-SFG	27	−9	72	−3.7132	57

### 3.4 Relationship between altered FC and clinical symptoms

**Figure 4:**
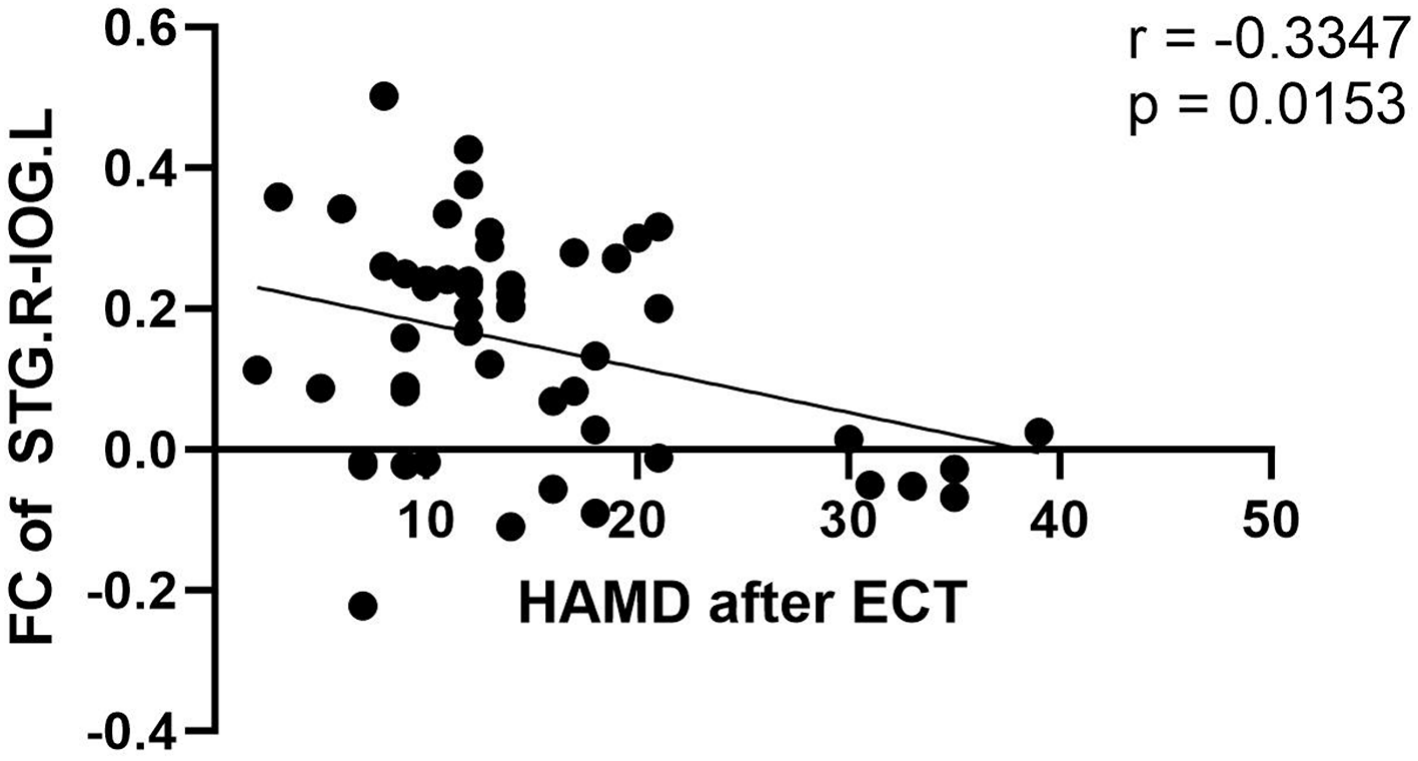
The negative correlation between HAMD scores after ECT and FC in R-STG and L-IOG.

Pearson correlation analysis: Using R-STG as ROI, FC between R-STG and L-IOG was found to be negatively correlated with HAMD scores after ECT. No correlation analyses were performed between BSSI scores and any FC measures in this study. Additionally, the correlation analysis between changes in clinical scores and changes in FC was not conducted due to the relatively modest sample size, which limits the direct verification of the association between ECT-induced FC alterations and the improvement of depressive or suicidal symptoms (Figure 4).

## 4 Discussion

This study designated L-SFG and R-STG as ROIs to compare baseline levels between HCs and MDDs, as well as pre- and post-ECT in patients. Based on whole-brain voxel-level FC analysis, it explored the efficacy mechanisms of ECT in adolescent MDD patients with SI. Findings revealed abnormal FC between R-STG and L-IOG in the MDD group when R-STG served as ROI, and abnormal FC between L-SFG and R-ACG when L-SFG was ROI. After ECT, FC from R-STG to R-MTG decreased. FC between L-SFG and R-MFG increased, and FC between R-SOG and R-SFG decreased. The lack of correlation analysis between changes in clinical scores and changes in FC further restricts the causal inference of ECT’s regulatory effect on brain circuits and clinical symptoms.

These findings suggest that abnormal FC within the frontal-limbic circuit may underlie the neurobiological basis of adolescents with MDD and SI. Dysfunction in these regions may lead to emotional bias, impaired executive control, and cognitive inflexibility, thereby increasing depressive symptoms and suicide/self-harm risk. ECT may exert its therapeutic effect by modulating FC within the frontal-limbic system and within key brain regions such as the frontal and temporal lobes, thereby alleviating depressive symptoms and suicidal ideation. The lack of correlation analysis between changes in clinical scores and changes in FC further restricts the causal inference of ECT’s regulatory effect on brain circuits and clinical symptoms.

Previous studies have extensively examined the relationship between suicidal symptoms in depression patients and prefrontal cortex activity. Baik et al. (2019) employed near-infrared spectroscopy to reveal that patients with MDD and suicidal ideation exhibited reduced activity in the left dorsolateral prefrontal cortex and left ventrolateral prefrontal cortex during verbal fluency tasks compared to HCs. Furthermore, changes in oxygenated haemoglobin levels within the left ventrolateral prefrontal cortex correlated with the severity of suicidal ideation. Willeumier et al. (2011) investigated cerebral blood perfusion in MDD patients with suicidal ideation, revealing reduced perfusion in bilateral superior frontal gyri. Oquendo et al. (2003) employed positron emission tomography (PET) to investigate alterations in regional cerebral metabolic rate for glucose (rCMRglu) in MDD patients with SA. They observed reduced rCMRglu in the superior frontal gyrus of the patient group, with rCMRglu levels correlating with the severity of suicide attempt symptoms. Based on these studies, we observe that the prefrontal cortex is involved in the development of suicidal symptoms. This mechanism arises from the prefrontal cortex’s role in emotional processing. Dysfunction in this region may impair an individual’s ability to accurately predict the consequences of negative emotions and detrimental decision-making, thereby increasing suicidal risk.

This study also identified abnormal FC within the frontal-cingulate circuit. The anterior cingulate cortex (ACC), a brain region responsible for emotional regulation and reward mechanisms, may experience dysfunction leading to emotional disturbances and subsequent suicidal symptoms. Neuroimaging studies indicate close relationship between the prefrontal cortex and ACC. Consequently, we hypothesise that disruption within the prefrontal-anterior cingulate circuit may contribute to suicidal tendencies in depression. Previous neuroimaging studies have suggested that female depressive patients with suicide attempts exhibit abnormal grey matter volume in prefrontal and limbic, indicating that alterations in the prefrontal-limbic circuit may influence decision-making biases, increase impulsive risk-taking, and consequently elevate the risk of suicidal behavior in this patient group (Moriguchi et al., 2014). Lungu et al. (2015) also identified the significance of prefrontal-limbic connectivity in emotional processing and task-related decision-making. Consequently, we consider that impaired internal connectivity within the front-cingulate circuit may elevate susceptibility to depressive symptoms and suicidal ideation. In this study, abnormal FC in this region among patients suggests that disrupted FC in the frontal-anterior cingulate circuit may remain a key mediator of depressive and suicidal symptoms in adolescents with MDD and SI. Pan et al. (2011) have examined the frontal-cingulate system in adolescents with depression and suicidality, finding higher activity in the frontal-anterior cingulate circuit among those with suicidal ideation when countering angry faces compared to depressed adolescents without such ideation. Overall, functional abnormalities in the frontal-anterior cingulate circuit appear to exert significant influence in adolescents with suicidal symptoms, warranting further investigation into the underlying neurobiological mechanisms.

We found notable alterations in intra-frontal FC following ECT, particularly involving the superior and middle frontal gyri. The middle frontal gyrus, a component of the prefrontal cortex, participates in memory and task execution processes. Taylor et al. (2004) examined elderly individuals with depressive disorders, noting a reduction in fractional anisotropy (FA) within white matter of left middle frontal gyrus. Bae et al. (2006) reported similar findings. Bremner et al. (1997) used PET imaging to demonstrate that even within general populations, depressive states may be associated with reduced metabolic activity in the medial frontal gyrus, these studies suggest a close association between medial frontal gyrus dysfunction and the depressive emotions.

Additionally, FC alterations were observed in the SFG and SOG, with the MOG recognised as crucial for processing emotional facial expressions. A meta-analysis examining FC in individuals with suicidal ideation revealed bilateral MOG alterations (Chen et al., 2022). Another study investigating depression and suicidal symptoms found reduced ALFF in left MOG in depressed patients with suicidal ideation compared to HCs (Fan et al., 2013). Research on ECT has examined alterations in this region.

Designating the R-STG as the seed, we identified abnormal FC between the R-STG and L-IOG in the MDD group at baseline. The STG is implicated in emotional and cognitive regulation, dysfunction in this region may contribute to cognitive inflexibility and impaired executive function, potentially leading to suicide-related symptoms. Functional imaging studies indicate that the superior temporal gyrus is associated with neural activity related to perception and memory (Gallagher and Frith, 2003; Saxe and Kanwisher, 2003), while also mediating processes predicting individual attempts and desire-driven behavioural capabilities. Pan et al. (2015) examined brain function in MDD patients with suicidal ideation, revealing enhanced ALFF activity in the R-STG compared to HCs. Structural MRI studies also link STG alterations to clinical symptoms, Fan et al. (2013) observed reduced grey matter volume in the right STG of adolescents with MDD and SA. Peng et al. (2014) similarly examined the relationship between temporal lobe volume and suicide risk, finding that abnormal grey matter volume of temporal correlates with SA. Therefore, we propose that abnormal structural and functional activity in the right STG may predict suicidal symptoms.

Following ECT treatment, alterations in FC between R-STG and R-MTG were observed. Functional imaging studies indicate these regions participate in psychological processes involving emotion, volition, and behavioural intent. Concurrently, the STG/MTG is implicated in emotional and cognitive regulation, as well as speech processing (Ellison et al., 2004; Karnath et al., 2001). Consequently, abnormal spontaneous brain activity in the STG/MTG may lead to emotional dysregulation, further mediating the emergence of depressive symptoms and increasing suicide risk. The potential mechanism of ECT may lie in regulating FC within the STG and MTG, ultimately improving depressive symptoms and suicidal ideation.

The FC modulation patterns observed after ECT in the present study may share some overlap with findings reported in other interventional neuromodulation studies, such as TMS, particularly in circuits involving frontal, temporal, and cingulate regions. This convergence may suggest that these networks are relevant treatment-responsive circuits in adolescent depression. At the same time, the current findings also raise the possibility that ECT may exert broader or partially distinct network effects, potentially related to its whole-brain mode of action. However, because the present study did not directly compare ECT with other interventions, any inference regarding treatment-specific mechanisms should be considered preliminary. Future head-to-head studies are needed to clarify the shared and distinct neural effects of different neuromodulatory treatments.

## 5 Limitation

This study employs rs-fMRI functional connectivity (FC) analysis, transitioning from region-of-interest (ROI) to whole-brain voxel-based methods. Defining the left superior frontal gyrus (L-SFG) and right superior temporal gyrus (R-STG)—regions previously identified as abnormal in our research—as seed points, we investigate alterations in FC among adolescents with major depressive disorder (MDD) accompanied by suicidal ideation (SI) at baseline and pre/post ECT treatment. Findings revealed abnormal functional connectivity in the R-STG and L-IOG, as well as in the L-SFG and R-ACG within the MDD group. Following ECT treatment, functional connectivity weakened from the R-STG to the R-MTG, while functional connectivity strengthened between the L-SFG and R-MFG. Functional connectivity between L-SFG and R-SOG, as well as R-SFG, diminished. These findings indicate that abnormal functional connectivity within the frontal-cingulate circuit contributes to the neurobiological processes in adolescents with MDD and suicidal ideation. ECT may ultimately improve depression with suicidal symptoms by enhancing internal functional connectivity within brain regions such as the frontal and temporal lobes.

The present study has several limitations that warrant consideration. First, the relatively modest sample size may have limited the statistical power and stability of analyses examining brain-behavior associations. Second, the lack of a non-ECT depressed control group prevents us from fully disentangling the independent effects of ECT from those of concomitant factors, such as repeated scanning and the passage of time. Third, our analyses were primarily focused on the association between depressive symptoms and ECT-related alterations in brain FC, without specifically exploring the direct relationship between suicidal ideation (assessed via BSSI scores) and treatment-induced FC changes, which constrains the depth of insights into suicide-related clinical outcomes. Fourth, the seed-based analysis was restricted to the L-SFG and R-STG, potentially overlooking other brain regions and networks that may be relevant to depressive and suicidal symptomatology. Fifth, the absence of long-term follow-up data precluded us from determining whether the observed FC changes were sustained over time or associated with longer-term clinical outcomes. Finally, all patients received continuous sertraline treatment during the ECT course, with a subset additionally administered aripiprazole; although an 8-week antidepressant washout period was implemented at baseline and the duration of medication exposure during ECT was relatively short, the potential influence of these medications on FC results should be acknowledged.

## Data Availability

The original contributions presented in the study are included in the article/supplementary material, further inquiries can be directed to the corresponding author.
